# Key Concepts in Dream Research: Cognition and Consciousness Are Inherently Linked, but Do No Not Control “Control”!

**DOI:** 10.3389/fnhum.2020.00259

**Published:** 2020-07-17

**Authors:** Caroline L. Horton

**Affiliations:** DrEAMSLab, Bishop Grosseteste University, Lincoln, United Kingdom

**Keywords:** sleep, dreaming, cognition, control, lucid dream

## Introduction

Whilst lucid dreaming (LD) is defined as being aware of dreaming whilst dreaming, a misconception exists in the public domain as a referral to controlling dream content and plot (Neuhäusler et al., 2018). This misconception reflects a number of widely-held beliefs about the nature of dreaming, which in part this commentary will seek to explain and rectify.

Furthermore, the aim of this piece is to suggest definitions of key concepts in the study of lucid and non-lucid dreaming concerning control, cognition, and consciousness. Whilst superficially there seems overlap between each of these, independent processes, and associated experiences underpin them.

### Dreaming

First it is necessary to identify the parameters of “dreaming.” Essentially dreaming refers to the recollection of mental content from sleep. This broad definition recognizes that dreams may be fragmented, brief, non-narrative, thought-like, and/or containing basic sensory-perceptual experiences such as emotions, without necessarily comprising complex plots or activity. It also emphasizes the role of memory in accessing experiences, as there are no valid means by which dreams can be sampled, as neither can individuals report on their activity during sleep nor can we independently validate individuals' experiences. Some scholars use “REM” (rapid eye-movement) sleep and “dreaming” synonymously (e.g., Walker, 2009), recognizing that the majority of spontaneously recalled dream reports emerge from REM sleep, and indeed that REM sleep provides the conditions most typical of dreams, such as bizarreness, clearer dream recall, emotionality and, likely, hyperassociativity (Horton and Malinowski, 2015; Malinowski and Horton, 2015; Horton, 2017), in which several distinct memory sources and images can be simultaneously experienced. However, dreams can be sampled easily from non-REM periods, and REM can exist without dreaming (Solms, 2000), thus is it essential to define the parameters of dreaming relevant to each scientific investigation. For instance, if we are interested in cognition and/or consciousness across different periods of sleep, or even across sleep and wake, then the term “mental content” or “mentation” may be more appropriate than “dream,” to aid such comparability (Kahan and LaBerge, 2011). If we are interested in characteristics such as emotional intensity or report length, then we need to clarify whether we should focus upon memory recall from sleep or the underlying features of a conscious state such as neurological correlates of such activity.

Next, for explorations LD, or even mere lucidity, researchers need to define and operationalise LD. An awareness of dreaming during dreaming relies on accurate reality monitoring processes (Johnson et al., 1984) as well as unbiased recall. Reality monitoring is typically impaired during sleep, hence making experiences of lucidity rare and interesting. However, in order to engage the frontal faculties sufficiently to warrant accurate reality monitoring, an atypical neurological profile is engaged (Voss et al., 2014). It is therefore important to note that lucidity is infrequent and abnormal (Vallat et al., 2018), and as such likely does not reflect “normal” cognition and consciousness during sleep, particularly when extensive training is necessary in order to create pre-requisite conditions for lucidity to emerge (e.g., Baird et al., 2019). Nevertheless, LD can be reliably measured, in laboratory conditions, by asking trained participants to move their eyes systematically whilst lucid (Mota-Rolim, 2020), and it is recognized that LD may provide insights into the nature of consciousness (Baird et al., 2019), albeit in a more artificial than naturally-occurring environment.

### The Elements of Cognition vs. Consciousness

As lucidity during sleep relies on heightened metacognitive activity, we need to understand what is meant by cognition during sleep and during wake. Cognition refers to the capacities and capabilities of function, in this case during sleep, in particular the organization, activation and reactivation of memories or experiences that are either familiar or unfamiliar to the dreamer. These processing capacities are notoriously difficult to study at any time, during sleep or wake, as some are so speedy they are automatic and operate beyond conscious awareness (see also the use of the term “offline processing” insofar as describing non-conscious cognitive activity, e.g., Wamsley, 2014). Consequently, it can be apparently tangible for researchers to focus upon the neural correlates of such behavior, to provide evidence for their functional existence (Baird et al., 2019). However, cognitive scientists need to offer theory for the function of such processes, for instance in relation to sleep-dependent memory consolidation (Payne and Nadel, 2004), rather than merely studying activations without considering functional relevance. In dream science, memory activations and predictable patterns of dreaming of familiar aspects of waking life have largely been explored under the Continuity Hypothesis (Schredl and Hofmann, 2003), as well as being observed in relation to other behaviors, such as personality traits (Schredl and Erlacher, 2004), moods, or subsequent performance on cognitive tasks such as problem solving, insight, creativity (Cai et al., 2009; Lewis et al., 2018), composition or recall (Baylor and Cavallero, 2001). Studies of cognition and metacognition during sleep have found that dreaming is not deficient but rather different in only a few ways to waking cognition (Kahan and LaBerge, 2011), with reality monitoring being one of the key different features. Specifically, during most sleep experiences, people cannot determine that their mental experience is internally- rather than externally-generated, consequently dreams feel real. Only in the cases of LD are individuals aware that they are dreaming. However, often the heightened metacognitive awareness is rousing and awakens the dreamer.

Whilst being aware of an experience as being internally- or externally-oriented can be operationalised in cognitive, or metacognitive terms, the conscious experience of that function may be characterized somewhat differently, although some features may overlap with those of cognition. Consciousness may, here, refer to the more characteristic features of sleep mentation, including experiential elements such as the fluidity, continuity over time, presence of specific features or characters and the more holistic nature of mental content. For instance, we may note that non-REM mentation is typically thought-like and brief, containing day residues and life-like references, whereas REM sampled mentation is typically bizarre, story-like and full of activity (Baylor and Cavallero, 2001; Blagrove et al., 2011). These descriptions of sleep mentation could well-reflect underlying cognitive processes such as memory activation, likely forming memory consolidation processes, but the overriding consciousness is more descriptive. The cognitive interests relate to function, and may be measures in those terms, such as extent of activation, which may also include aspects that are non-conscious at the point of experience.

When considering lucidity, the nature of the consciousness may include sensations of awe at realizing one is dreaming, as well as vivid memories of the dream experience itself. This is commonly associated with increased underlying neurocognitive activity. The underlying *cognition*, or hypothetical function, reflects accurate reality monitoring, metacognition, self-awareness and, typically, arousal (from enjoyment of the experience).

Furthermore, in some studies of LD, participants who achieve lucidity may continue to develop the ability to control their actions during dreaming (LaBerge, 1980). Indeed, several studies aimed to achieve this, rather than studying the mere presence of lucidity in more naturalistic or opportunistic settings. Such studies confuse the concepts of lucidity and control, with the former being more likely to occur naturally, and the latter being rare and artificial experiences. As such scholars should be cautious about inferring the nature of consciousness and/or cognition from artificial control-induction techniques, as this likely differs from the profile of mental content emerging from experiences of lucidity.

LD is unusual, relative to the existence of dreaming which, arguably, occurs the entire time that one is asleep (if the present definition of dreaming is adopted, as consciousness continues, even during sleep). Whilst lucid, or controlled, experiences may offer a therapeutic benefit, for instance by allowing individuals to rehearse actions (Stumbrys et al., 2016) or overcome threats (Putois et al., 2019) during sleep, they are typically fleeting, and estimations of their frequency often rely on self-report and retrospective methods (Vallat et al., 2018). Furthermore, inducing lucidity interrupts sleep, which we know is required to facilitate emotion-regulation and memory consolidation processes, which arguably would be more beneficial than any benefits of lucid dreaming anyway (Vallat and Ruby, 2019).

### Control

To operationalise lucidity, researchers should take care not to confuse controlling the dream experience with mere awareness of dreaming. We should then define control carefully for instance as voluntarily changing experience. Superficially control may seem to rely upon both a specific cognitive and consciousness profile, however the conscious awareness of control may only become apparent at the time of recall, rather than during the experience itself, and again scholars should take care to identify any potential additional explanatory information offered to a dream report at the point of reporting it, as being distinct from a description of the original experience.

Caution should be urged when considering whether it may be appropriate to recommend that participants control their dreams, given that doing so increases sleep disturbances via awakenings (however, see LaBerge et al., 2018a, who included data from uninterrupted REM sleep only, but see also LaBerge et al., 2018b, for a paradigm in which participants remained awake for 30 min in the middle of the night, which increased LD recall), and also that controlling dream content is unnatural, therefore it may restrict the activation of memory sources and emotions that may underly sleep-dependent memory consolidation (Wamsley and Stickgold, 2011) and emotion regulation (Walker, 2009) processes. Perhaps only in the case of nightmares causing substantive distress, most typically in sufferers of post-traumatic stress disorder, should the possible benefits of reducing distress from terrifying dreams outweigh the likely negative consequences of changing sleep structure and physiology, by restricting the opportunity for “offline” processing (e.g., Putois et al., 2019).

In the occasions of spontaneous ongoing lucidity, whereby the experience does not awaken the dreamer, either the dreamer attempts to understand, or even “interpret” meaning from the typically bizarre dream narrative in which they find themselves, or they attempt to control it in some form during the dream state. The latter, in the case of LD, can be learned in some cases (LaBerge, 1980). Comparable practices during wakefulness demonstrate the ability for some to being able to gain fuller awareness of some typically more automatic behaviors, as depicted by the rise in popularity of mindfulness.

LD is concerning for a number of reasons, as recently outlined by Vallat and Ruby (2019), whereby training to overcome the mental content spontaneously emerging during sleep-dependent cognition ultimately changes and thwarts those processes. Humans likely need to foster the conditions for those processes to occur in order to benefit from the plethora of advantages of sleep.

It seems surprising that LD has received much attention, when time spent dreaming is far greater. Furthermore, the nature of dreaming and consciousness is fascinating, and may provide insights into the nature and perhaps function of underlying cognitive processes. For instance, dream bizarreness, which typifies REM mentation (Revonsuo and Tarkko, 2002; Payne, 2010) and likely results, at least in part, from hyperassociativity of distinct memory sources during sleep (Horton and Malinowski, 2015) may inform an understanding of the activation, fragmentation and re-organization of memory sources as part of sleep-dependent memory consolidation processes (Horton, 2017). Lucidity, however, is highly atypical and therefore arguably cannot offer so much insight.

## Discussion

“Control” within LD inherently unnatural and disrupts sleep. Controlled dreams rarely exist spontaneously, either in typical or atypical cognition. Scholars therefore should have the integrity to consider the impact that studies of control may have not only on participants engaging with such studies, but also the wider community who may be attracted to the idea of controlling their dreams. There is a duty to convey that we should not control, control, but instead promote the benefits of sleeping well (Walker, 2019), to afford the opportunity to dream.

Nevertheless, it is important to consider whether LD may have adaptiveness value, especially in the case of emotion processing and/or when the incidence of LD correlates with pathologies. LD may also provide insights into the nature of dreaming, principally by involving the dreamer during the dream (Zink and Pietrowsky, 2015), rather than just afterwards during recall.

## Author Contributions

The author confirms being the sole contributor of this work and has approved it for publication.

## Conflict of Interest

The author declares that the research was conducted in the absence of any commercial or financial relationships that could be construed as a potential conflict of interest.
